# Figure-ground perception in the awake mouse and neuronal activity elicited by figure-ground stimuli in primary visual cortex

**DOI:** 10.1038/s41598-018-36087-8

**Published:** 2018-12-12

**Authors:** Ulf H. Schnabel, Christophe Bossens, Jeannette A. M. Lorteije, Matthew W. Self, Hans Op de Beeck, Pieter R. Roelfsema

**Affiliations:** 1 Department of Vision & Cognition, Netherlands Institute for Neuroscience, Meibergdreef 47, 1105 BA Amsterdam, The Netherlands; 2 Laboratory of Biological Psychology, Brain & Cognition, KU Leuven, Leuven, Belgium; 3 Cognitive and Systems Neuroscience Group, Swammerdam Institute for Life Sciences, Faculty of Science, University of Amsterdam, Amsterdam, The Netherlands; 4 Psychiatry Department, Amsterdam University Medical Center, University of Amsterdam, Amsterdam, The Netherlands; 5 Department of Integrative Neurophysiology, Vrije Universiteit, Amsterdam, Amsterdam, Neuroscience, The Netherlands

## Abstract

Figure-ground segregation is the process by which the visual system identifies image elements of figures and segregates them from the background. Previous studies examined figure-ground segregation in the visual cortex of monkeys where figures elicit stronger neuronal responses than backgrounds. It was demonstrated in anesthetized mice that neurons in the primary visual cortex (V1) of mice are sensitive to orientation contrast, but it is unknown whether mice can perceptually segregate figures from a background. Here, we examined figure-ground perception of mice and found that mice can detect figures defined by an orientation that differs from the background while the figure size, position or phase varied. Electrophysiological recordings in V1 of awake mice revealed that the responses elicited by figures were stronger than those elicited by the background and even stronger at the edge between figure and background. A figural response could even be evoked in the absence of a stimulus in the V1 receptive field. Current-source-density analysis suggested that the extra activity was caused by synaptic inputs into layer 2/3. We conclude that the neuronal mechanisms of figure-ground segregation in mice are similar to those in primates, enabling investigation with the powerful techniques for circuit analysis now available in mice.

## Introduction

An important first step in the analysis of a visual scene is the segregation of figures from the background. The successful segregation of the image into figure and ground is essential for the identification and localization of objects. Most research on the neural mechanisms underlying figure-ground segmentation used the non-human primate as a model to study the activity of cortical neurons^1–4^ and fMRI in humans to investigate more global patterns of neuronal activity across the different cortical areas^5,6^. Previous studies proposed that figure-ground segregation relies on feedback loops between lower and higher areas of the visual cortex^2,7–9^. Figure-ground segregation could therefore be a useful paradigm to investigate how neurons in different cortical areas interact with each other during visual perception.

**Figure 1:**
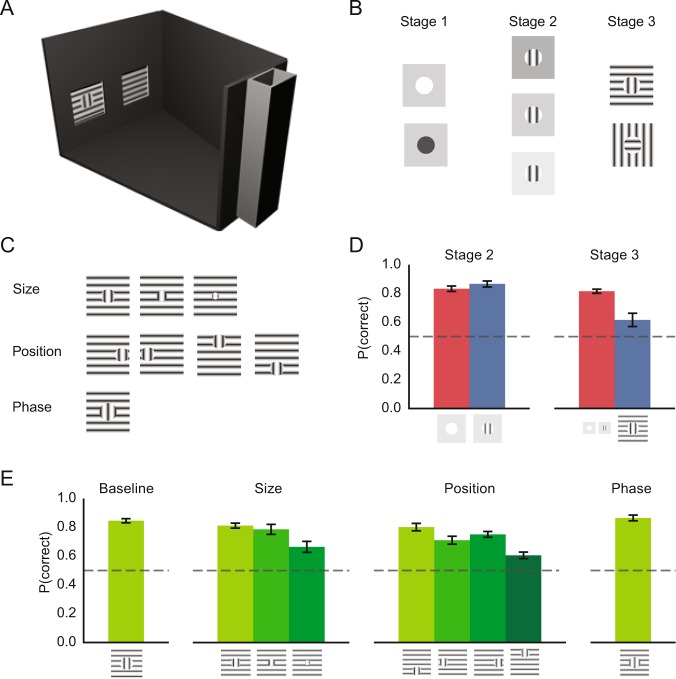
Figure-ground perception in mice. (**A**) Schematic of the setup for the behavioral experiment, with a feeding box on the right and two touch screens on the left. (**B**) The mouse had to choose between a stimulus with a circle (the figure) and a homogeneous display without a circle. Reward was given if the animal chose the stimulus with the figure. During stage 1 of the training process, the mice saw circles that were either lighter or darker than the background. During stage 2 we presented a grating stimulus on a homogeneous background and during stage 3 we included grating figures superimposed on a background with the orthogonal orientation. (**C**) For the tests of generalization, we either varied the size, the position or the phase of the figure. Note that the grating orientation of the figure and background could be either vertical or horizontal so that the local orientation could not be used to solve the task. (**D**) Accuracy for the familiar (red bar) and new (blue bar) stimuli in the first sessions of stage 2 and stage 3. Error bars indicate SEM. In stage two, the luminance defined circles were replaced by gratings. Each of the seven mice performed 40 familiar and 60 new trials in stage 2. In stage 3, the mice saw figure-ground stimuli with a figure grating on top of a background with the orthogonal grating for the first time. Here each mouse performed 80 familiar and 28 new trials. Example stimuli are shown underneath each bar. (**E**) Average baseline accuracy for the figure-ground (stage 3) stimuli and accuracy in the generalization tests. Each mouse performed 100 trials for the baseline condition and 60 trials for each of the other conditions. Error bars indicate SEM across 7 animals.

Figure-ground perception involves a number of successive processing steps^3,10,11^ (see Fig. 1B “stage 3” for example figure-ground stimuli): (1) An initial feedforward input from the retina drives visual responses in low-level and higher level visual areas. During this phase the excitation of cells mainly relies on AMPA receptors and neurons represent the features in their receptive fields^1–4^. (2) In the next phase, neurons are also influenced by information outside their receptive fields. The edges between figure and ground start to elicit extra activity, suggesting a process that is sensitive to the feature contrast at the boundary between figure and background. This extra activity occurs selectively in the supragranular layers^12^. (3) The next phase is region filling. Now the representation of all figural image elements is enhanced in V1, including the representation of figure center. This region-filling phase relies on a feedback signal from higher areas to lower areas^13,14^, depends on NMDA receptors, and is most pronounced in the supragranular and infragranular layers^12^. A TMS study in humans suggested that disruption of this late phase in early visual cortex interferes with figure perception^15^ (for a recent review of the different phases of the V1 activity for the computation of the figure-ground percept see ref.^14^).

Previous studies demonstrated that rats exhibit size-invariant and translation-invariant object-recognition^16^ and that they can segregate figures from backgrounds on the basis of a difference in orientation^17^. Figure-ground perception has, to our knowledge, not yet been studied in mice. It would be advantageous to also be able to study figure-ground perception in mice, because this approach could benefit from the powerful methods that now exist to investigate the circuitry and neuronal activity at the systems, cellular and subcellular level in this species^18,19^. One earlier study in the primary visual cortex (V1) of anesthetized mice investigated the activity elicited by a figure of one orientation superimposed on a background of the opposite orientation^20^. The figure evoked more V1 activity than the background. However, in the anesthetized mouse figure-ground modulation was strongest in layer 4 and the superficial layers, whereas in the monkey it is most pronounced in the superficial layers and deep layers but relatively weak in layer 4. It is possible that the absence of figure-ground modulation in the deep layers of V1 of mice was caused by anesthesia, because anesthesia also blocks figure-ground modulation in monkey V1^21^. Furthermore, it is unknown if mice can perceive orientation defined figure-ground stimuli, although many aspects of mouse behavior, such as hunting for insects^22^ and the detection of objects passing overhead^23^ are likely to depend on figure-ground perception. In the present study, we therefore investigated whether mice can perceive orientation-defined figure-ground stimuli and we examined the neuronal activity elicited in the different layers of V1 of awake mice.

## Methods

We report two experiments. The first is a behavioral experiment to assess the ability of mice to detect figures of one orientation on a background of the opposite orientation. The second is an electrophysiological experiment where we measured the response of neurons in the different layers of V1 of awake mice elicited by these figure-ground stimuli.

### Behavioral experiments

#### Animals

We tested eight C57BL6/J mice, which were housed in two cages of four mice each. Each cage was enriched with cardboard material. The mice were approximately 10 weeks old at the start of the experiment. Access to food pellets was restricted to approximately one hour each day, immediately following the experimental session. We measured the body weights of the animals daily to make sure that no animal dropped below 85% of their initial free feeding weight. All procedures were approved by the KU Leuven Animal Ethics Committee and all experiments were performed in accordance with the relevant guidelines and regulations.

#### Visual stimuli

The training proceeded in three stages as will be described below. In the final test, the mouse had to discriminate between a grating stimulus with a circular figure and a homogeneous textured background by touching one of two screens. Fig. 1A,B provides an overview of all stimuli that were used to train the animals in the behavioral experiments. The stimuli were constructed from sine-wave gratings at maximum contrast (black pixel luminance 22.1 cd/m², white pixel luminance 298 cd/m², measured with Minolta a CS-100A Chroma Meter). The target stimulus contained a circular figure with a vertical or a horizontal orientation that was superimposed on a background grating with the orthogonal orientation. At a distance of 5 cm from the screen, the spatial frequency of the grating was 0.08 cycles per degree and the diameter of the circle in the behavioral experiments was 30°. This distance is a conservative estimate of the position at which animals decide on their response, as the touch screen set-ups did not provide exact control over the distance between the mice and the screen at the decision time. We varied the stimuli to investigate how well the mice could generalize figure detection to new stimulus configurations (Fig. 1C). We changed the size of the circle figure (decreasing its size to 24°, 18° and 12°). We also tested different positions of the 18° figure (shifting it to the top, bottom, left or right of the screen), positions that did not overlap with the original position. In other experiments, we changed the phase of the circle texture by 180°.

#### Setup and operant procedures

We trained the mice in touch screen operant chambers (Campden Instruments Ltd., Leicester England), which were placed in a sound-attenuating box. On one side of the chamber was a touch screen monitor with a Perspex mask with two square extrusions that were used for stimulus presentation (Fig. 1A). On the opposite side was a reward tray, which delivered strawberry milkshake as reward.

We first made the animals familiar with the behavioral protocol through a standardized shaping procedure^24^. During this procedure, the mice learned to discriminate black and white circles (50% black and 50% white, chosen at random) on a homogeneous grey screen (Fig. 1B, stage 1), i.e. they had to touch the screen that displayed the circle. In this phase, they learned that a correct response was associated with a reward. Then they learned to initiate a trial by nose poking in the reward tray and that an incorrect response was associated with a time-out.

The actual experimental protocol started after this shaping procedure. Each animal participated in a single experimental session each day, which consisted of approximately 50 trials (the precise number depended on the type of stimuli that were presented) and lasted 1 hour. The beginning of a trial was signaled by the illumination of the reward tray. If the animal put its head in the reward tray, the light was extinguished and the stimuli were presented on the touch screens. Hence, the stimulus onset occurred when the animal did not face the stimuli. Then the animal had to approach one of the touch screens and it received the reward if it touched the correct stimulus. After collecting this reward, there was an intertrial interval of 20 seconds before the next trial could be initiated. If the animal touched an incorrect stimulus, a house light was illuminated for 5 seconds followed by an inter-trial interval of 20 seconds. After this time-out period, the stimulus of the previous trial was presented again until the animal made a correct response. All trials following the first incorrect response were labeled as correction trials and they were not taken into account for the analysis.

The training procedure had three stages (Fig. 1B) and the animals reached the next stage when their performance was above 80% correct on two consecutive sessions. During the first stage, the difference between figure and background was defined using luminance cues only. The circle figure could be either lighter or darker than the background, ensuring that animals could not base their decisions on the average luminance. The second stage included target circles filled with gratings against a uniform background. The luminance of the background varied across trials so that the average luminance of the circle figure was sometimes higher and sometimes lower than the background. In stage 3 we added grating stimuli on an orthogonal grating background. During this stage, we also showed the stage 1 and 2 stimuli, while figure-ground grating stimuli were shown in 44% of the trials. A subset of animals had difficulties acquiring criterion performance of 80% in stage 3 and for these animals we switched to sessions where only the grating-defined figure-ground stimuli were presented. This stage 3 paradigm was also used as a baseline for each of the transfer tests described next. Importantly, we always made sure that animals performed above criterion performance on the baseline test before proceeding to the next stage.

Transfer of learning was assessed for size, position and phase (Fig. 1C). For each of these variations a session included both the stimuli from the baseline test (50% old stimuli) and the new stimuli (the other 50%). In the size variation test, we decreased the stimulus size from 30° to 12° in steps of 6° (only one of the new sizes was used in every session) and, starting from the smallest stimulus size, we then increased the stimulus size again so that each animal participated in two sessions per stimulus size. The influence of stimulus position was assessed in sessions where the target circle was displaced to one of four possible locations: left, right, above or below the original location. We performed two sessions per location per animal. As before, these trials were intermixed with an equal number of trials with the stimulus at the original location. Sensitivity to phase was assessed by applying a 180° phase offset to the figure grating. In the analysis of behavior, we will report the performance across two sessions per animal, but an analysis of only the first session produced comparable results.

#### Electrophysiological recordings in the visual cortex of mice

**Figure 2:**
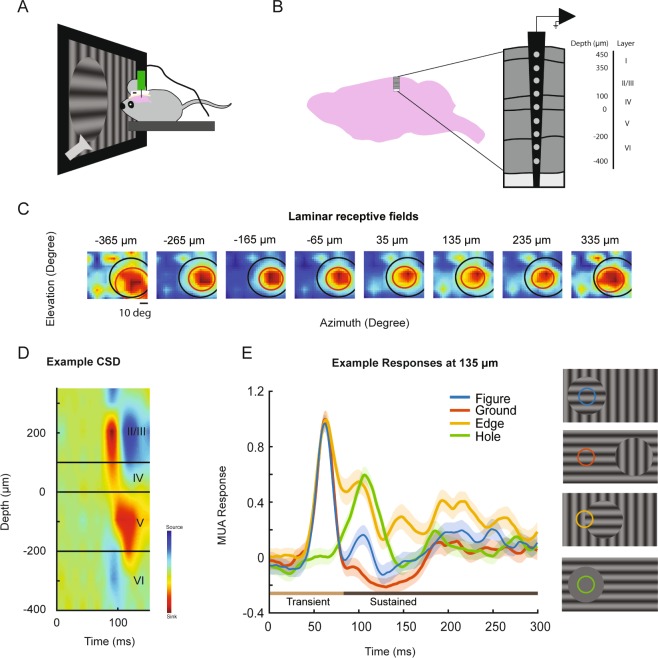
Electrophysiological recordings. (**A**) For the V1 recordings, the mouse’s head was immobilized in front of a screen and we presented visual stimuli while using a laminar probe to record V1 activity. Eye position was monitored with an eye tracker. (**B**) We inserted a laminar electrode with a spacing of 100um between the recording sites into primary visual cortex (**C**) RFs of the different recording sites of an example penetration (see Methods). Red ellipses represent the full width at half maximum of a 2D-gaussian fitted to the response profile to estimate the RFs. Black line indicates the perimeter of the figure stimulus. (**D**) CSD of the same penetration. The bottom of the early sink demarcates the boundary between L4 and L5. This sink provides an estimate of the position of the electrode and was used to align the data of different penetrations. (**E**) MUA responses elicited by the visual stimuli (illustrated on the right) at one example recording site in layer 2/3. The color of the traces corresponds to the color of the receptive field on the visual stimuli shown on the right. The shaded area shows the SEM across trials.

We recorded from area V1 in four male adult C57BL6/J mice while they viewed visual stimuli but the animals did not perform a task during these recording sessions. The experimental procedures complied with the National Institutes of Health Guide for Care and Use of Laboratory Animals, the protocol was approved by the ethical committee of the Royal Netherlands Academy of Arts and Sciences and all experiments were performed in accordance with the relevant guidelines and regulations. All mice were implanted with a head post under 2% isoflurane anesthesia and antiseptic conditions to allow head immobilization. Before the surgery, we applied local (xylocaine to the skin before the incision) and systemic analgesia (Metacam, subcutaneous, 2 mg/kg). After induction of the anesthesia, the frontal part of the skull was exposed and thoroughly cleaned. We then fixed a plastic head post to the skull with dental cement. After five days of recovery, we started with head immobilization training. We started by immobilizing the head of the mice in their home cage until they stayed calm (usually 3–5 sessions). The next step was to put the mouse in the setup (Fig. 2A) for increasing periods of time until they were habituated to sitting in the setup. We placed a tube on top of the mouse to restrict movements and provide a comfortable resting place, which they accepted within one or two sessions. Once the mice were comfortable in the setup, they underwent a second surgery under isoflurane anesthesia. We created a small craniotomy with a diameter of approximately 0.8 mm in the skull above V1 (3 mm lateral and 0.4 mm anterior of lambda) and formed a chamber around the craniotomy using dental cement, which we sealed using silicone and bone wax. The chamber was cleaned once per week and after a recording. If necessary, growing tissue and bone were removed under brief isoflurane anesthesia to keep the chamber open.

#### Recording of multi- and single unit activity

We recorded neuronal activity (Fig. 2B) in area V1 using a 16-contact laminar electrode (Neuronexus A1x16–100mm-100-43) with a 100 µm spacing between contacts. The electrical signal was amplified and digitized at 24.4 kHz by a system manufactured by Tucker-Davis-Technologies. From this signal, the LFP was extracted using a low pass filter with a corner frequency of 200 Hz. The envelope of the multi-unit activity (MUAe) was extracted using a band pass filter with corner frequencies of 500 Hz and 5000 Hz. The data was rectified and low pass filtered at 200 Hz and sampled at a frequency of 763 Hz^25^. Spikes of individual neurons were detected using a variable voltage threshold at approximately 2 standard deviations (SDs) of the signal that was set during the recording. Thirty samples of the spike waveform were saved at the original sampling frequency for later spike-sorting analysis. We isolated 85 cells, of which 82 reached a firing rate of between 2 Hz and 12 Hz during the initial transient response and three cells had a firing rate larger than 20 Hz. We tracked the pupil of the eye contralateral to the recording site using an ISCAN system at a sampling frequency of 120 Hz.

#### Visual stimuli

We presented different stimuli during successive phases of a recording session on a 21-inch LCD screen with 1280 × 720 pixel resolution (DELL 059DJP) driven at 60 Hz by a windows computer. The screen was positioned in front of the mouse at a distance of 11 cm. First, we presented a checkerboard of bright (luminance 10.3 cd/m^2^) and dark (luminance 0.01 cd/m^2^) squares (edge length 10°) to probe the visual response and to record an initial current source density (CSD) profile to verify and adjust the depth of the laminar probe on-line. Second, we presented flashing bright (luminance 44 cd/m^2^) squares (edge length 10°) covering an area of the visual field ranging from −60° to 20° horizontal and −30° to 30° vertical relative to the mouse’s nose in the hemifield contralateral to the V1 penetration to map the neurons’ receptive field (RF). The RF was calculated on-line and the recording was aborted if there was no clearly distinguishable receptive field or if it was too close to edge of the screen. We fit the response profile with a 2D-Gaussian to determine the position of figure-ground stimuli. Third, we presented the figure-ground displays (Fig. 2E, right). The figure-ground stimuli consisted of a static figure, a circular patch of sinusoidal grating (80% contrast, spatial frequency of 0.075 cycles/deg), with a size of 50° superimposed on a background grating of the orthogonal orientation that filled the screen. We chose a size of 50° to ensure that the edge of the figure did not fall in the neurons’ receptive fields, except in one of the conditions (see below). The ratio between figure-size and receptive field size was approximately 1:2, which is slightly larger than the 1:4 ratios used in previous studies in monkeys (in monkey V1 parafoveal RFs are approximately 1°)^2,3,21^. We placed the figure so that the RF of neurons in layers 2/3 would fall in the figure center. In two out of 29 cases, the figure touched the edge of the screen and a small fraction of it fell outside the screen, but even in these cases most of the figure was visible and surrounded by the background grating. There were a total of four stimulus conditions that were each presented in 25% of the trials (Fig. 2E, right). In the “figure” condition the center of the circular foreground grating fell in the RF, as was described above. In the “edge” condition the grating was shifted by 25°, so that the edge between figure and background fell in the RF. In the “ground” condition we presented the circular figure in the opposite hemifield, on the other side of the screen. Finally, in the “grey hole” condition we placed a 50° grey circle at the figure position on the background grating. All stimuli were presented for 0.5 s with an inter-trial interval that varied between 1 s and 1.5 s. We presented a figure with a horizontal orientation on a background with a vertical orientation or vice versa, so that the orientation of the grating in the receptive field was, on average, the same in the figure and background conditions (see red and blue RFs in Fig. 2E for stimulus conditions with identical RF stimulation).

#### Data Analysis

*Current-source density (CSD) analysis*: We used the CSD to estimate how deep the electrode was inserted into the cortex by measuring the laminar location of current sources and sinks^12,26–29^. It is calculated by taking the second spatial derivative of the LFP using the following formula:

1 $$CSD(x)=-\sigma \cdot \frac{\phi (x-h)-2\phi (x)+\phi (x+h)}{{h}^{2}}$$

Where *φ* is the voltage, *x* is the point at which the CSD (in A.mm^−3^) is calculated, *h* is the spacing of recording sites used for the computation (here 0.2 mm) and *σ* is the tissue conductivity (we used 400 S.mm^−1^)^30^. We calculated the CSD based on the response elicited by the checkerboard stimulus, and used it to find the current sink in layer 4 of V1. We used the lower boundary of this sink as an estimate for the border between layers 4 and 5 and assigned a depth of zero to this position. Before averaging, we aligned different penetrations using this position and hence used it as reference point, specifying the depth of other contacts relative to this location. The recordings spanned approximately 700 µm. We assigned the shallowest 200 µm to Layers 2/3, followed by 100 µm of Layer 4 and we split the next 400 µm evenly into Layers 5 and 6. The significance of the CSD profiles was determined using clustered t-tests, as described in ref.^12^.

*Movement rejection:* In some of the trials we observed large artifacts in the electrophysiological data that were caused by motion of the mouse. We identified such trials by visual inspection and removed them from further analysis. Additionally, all trials in which the eye of the animal moved out of its resting position were removed from analysis. We calculated the Euclidian distance from the median eye position during every trial, computed z-scores based on the distribution of distances across samples and removed all trials in which the z-score reached a value larger than 1.5. This lead to the removal of 21% of trials.

**Figure 3:**
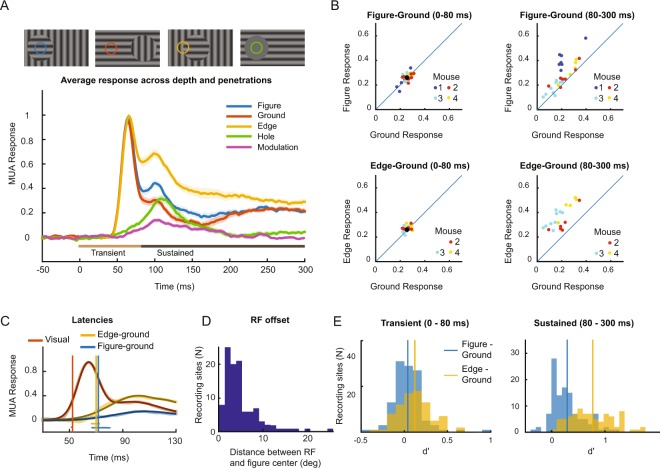
Multi-unit activity elicited by the figure-ground stimuli in V1. (**A**) The V1 MUA response elicited by the different stimuli, averaged across all layers of the 21 penetrations for which all four stimuli were presented. The shaded area indicates the SEM across penetrations. (**B**) Comparison of activity elicited by the figure and background (top) and the edge and background (bottom) during the early (0–80 ms, left) and late time-window (80–300 ms, right). Data from the different mice are shown in distinct colors. Each data point represents the average activity across all recording sites of one penetration. (**C**) The latency of the visual response and the figure-ground modulation was determined with a curve-fitting method (see Methods). Thick lines show the data and thin black lines the fits. The vertical lines show the estimated latency of the visual response (red), the difference between the edge and background response (yellow) and the difference between the figure and background response (blue). The horizontal lines indicate the 95% confidence interval of the latencies as determined with a bootstrapping method. The red horizontal line is difficult to see because the 95%-confidence interval is very narrow. (**D**) Distribution of the distance between the center of the RFs of the MUA recording sites and the figure center. (**E**) Distribution of d′ values of the MUA for discriminating between figure and ground (blue) and between edge and ground (yellow) during the early, transient (0–80 ms, left) and late, sustained time-window (80–300 ms, right).

*Normalization and statistics:* The MUAe response was averaged over all trials of each condition, separately for every channel. MUA channels with a signal-to-noise ratio (SNR; visual response divided by the standard deviation of pre-stimulus activity in a 200 ms window before stimulus onset, across trials) below 2 were excluded. The average pre-stimulus activity (MUA in the 200 ms time-window before stimulus onset) was then subtracted, and the responses were divided by the maximum of the response to the background grating for normalization. We defined two time-windows for the analysis of the strength of the visual response. The first time-window included the transient response: 0–80 ms after stimulus onset. The second time-window quantified the sustained level of activity once the transient response was over, from 80–300 ms after stimulus onset (light and dark brown horizontal bars in Fig. 3A).

*d*′ *calculation:* We calculated the d′ as the difference in the mean response between figure and background, divided by the pooled standard deviation, i.e.:

2 $$d^{\prime} =\frac{{\mu }_{Fig}-{\mu }_{Grnd}}{\sqrt{0.5\,\ast \,({\sigma }_{Fig}^{2}+{\sigma }_{Grnd}^{2})\,}}$$

*Latency estimation:* To estimate latencies of the visual response or the response modulation, we fitted a curve to the population response^12^ and estimated the latency as the time point when it reached 33% of it’s maximum. We performed a bootstrapping procedure to calculate the 95% confidence intervals and to test for significance.

*Statistical testing:* We ran two-sample and paired t-tests where appropriate. ANOVAs had a repeated measure design and were corrected according to Greenhouse-Geisser if sphericity assumptions were violated. We performed Bonferroni corrected post-hoc comparisons of conditions.

*Single-unit activity:* The recorded spikes were clustered offline using the WaveClus toolbox^31^ according to their size and shape waveform. Only clusters with distinct waveforms, no more than 3% of inter-spike intervals below 3 ms, an evoked response stronger than 2 Hz, and a signal-to-noise ratio above 1.5 were included as single units.

## Results

### Behavioral task

A total of eight mice were trained to test whether they can perceive orientation-defined figure-ground displays. Seven mice learned the task, but one animal failed to acquire the stimuli of stage 1 (luminance-based figure detection, Fig. 1B) and was still performing around chance after 49 sessions. Data from this animal was therefore not taken into account and all analyses are based on the results from the 7 other animals. These animals took 9.1 (SEM = 1.5) sessions to master the stage 1 discrimination task based on luminance only (Figure S1). Introducing the circular grating in stage 2 had no effect on performance (Fig. 1D, left). Mean performance for the luminance-defined stimuli, which had already been introduced at an earlier stage, was 83% (SEM = 1.9%) and the accuracy for the new grating stimuli was 86% (SEM = 2%). This difference in accuracy was not significant (paired t-test with 6 degrees of freedom, t(6) = −0.942, p = 0.382).

In stage 3, we introduced the figure-ground stimuli. At the start of this stage, the animals did not immediately transfer to grating circles with one orientation on backgrounds with gratings of the orthogonal orientation (Fig. 1D, right). In the early sessions, the mean accuracy for the now familiar stage 2 stimuli was 81% (SEM = 1%) but it was only 61% (SEM = 5%) for the new grating defined stimuli (t(6) = 3.7, p = 0.01). Two animals reached criterion performance (80% or better) after 8 sessions. The remaining animals were transferred to sessions in which we only presented the stage 3 stimuli (see Methods and Figure S1). Now they did reach criterion performance after an average of 3.4 sessions (SD = 1.3). The leftmost bar in Fig. 1E shows the final accuracy for the stage 3 stimuli, averaged across the last two sessions of the 7 animals.

We then conducted three tests to see how learning transferred to variations of the figure-ground stimuli. First, we tested the influence of figure size by measuring the accuracy for figures of 12°, 18° and 24° (Fig. 1E, second graph), which were smaller than the original figures of 30°. The accuracy was lower for the smaller figures (one way repeated measures ANOVA, F(2,12) = 5.3, p = 0.022). A paired t-test revealed that accuracy with 12° figures was lower than that with 24° figures (t(6) = 2.7, p = 0.035), but for each size the accuracy remained above chance level (all ps < 0.01). Thus, the mice were able to generalize their performance across a considerable variation in the size of the figures.

A change of the figure position also influenced performance (F(3,18) = 12.1, p < 0.001) (Fig. 1E, third graph). Specifically, performance for the top position was lower than that for the other positions (all ps < 0.025), but the accuracy was above chance for all positions (all ps < 0.01). Thus, animals did not show perfect generalization when the circle figure was not in the original position, but they nevertheless generalized to all positions that we tested. Finally, the accuracy for the stimulus in which the phase of the figure grating was shifted by 180° (Fig. 1E, right) was above chance level (t(6) = 16.9, p < 0.001) and did not significantly differ from that for the stimuli with the original phase (t(6) = 1.24, p = 0.26).

### V1 activity elicited by figure-ground stimuli in awake mice

We next investigated the processing of figure-ground stimuli in area V1 of 4 awake mice and recorded neuronal activity with laminar probes with a spacing of 100 μm between contact points for a total of 29 penetrations (Fig. 2B). We first mapped the receptive fields of the MUA at the different depths. The red ellipses in Fig. 2C illustrate the overlapping RFs of one example penetration, in accordance with a positioning of the electrode perpendicular to the cortical surface. We next determined the CSD (Fig. 2D) to evaluated the placement of the electrode in the cortical layers. The CSD had a characteristic sink in layers III-IV (red in Fig. 2D)^12,26–29^ and we confirmed that the electrode covered all the layers of primary visual cortex.

We then presented the figure-ground stimuli. The figure was a round, 50° region with an orientation that was orthogonal to the background orientation (Fig. 2A; black circle in Fig. 2C). We presented a figure with a horizontal orientation on a vertical oriented background on some trials and used the opposite combination of orientations on other trials. We thereby ensured that the grating in the RF was exactly the same in the figure and background conditions and that only the context was different. Thus, differences in activity elicited by the figure and background condition have to be attributed to differences in the context, determined by the stimulus outside the classical RF. In figure trials, the RFs with an average size of 30° fell fully within the figural region of 50°. We also included an “edge” condition, in which we placed the edge between the figure and the background in the receptive field. The final stimulus was a “hole” condition where the grating stimulus only appeared outside the RF while nothing changed within the RF upon stimulus presentation (Fig. 2E, right).

Figure 2E illustrates the V1 MUA response at one of the recording sites of the example penetration, at a depth of 135 μm in Layer 2/3. The amplitude of the initial response was similar in the figure, ground and edge conditions (time window 0–80 ms after stimulus onset, all Ps >0.05). However, after a delay the response to the edge became stronger than that to the figure, which, in turn, became stronger than the response to the background (time window, 80–300 ms, ANOVA F(3,195) = 22.1, p < 0.001). Post hoc tests revealed that activity elicited by the figure center was stronger than that elicited by the background, and that activity elicited by the edge was stronger than that elicited by the figure center (p < 0.05). Surprisingly, in the “hole” condition nothing appeared in the RFs, but the neurons exhibited a delayed increase in their activity (green in Fig. 2E). The timing of the activity elicited by the hole stimulus corresponded to the timing of the response enhancement elicited by the figure or edge relative to the activity elicited by the background.

The results that we obtained for this example recording site were representative for the effects across all penetrations. The average size of the RFs of the MUA recording sites was 29.5 degree (full width at half maximum of 2D Gaussians that were fitted RF profiles, see Methods). Across the population, the RFs were well centered on the figure, with an average distance between the RF-center and the figure center of 5.2 degrees and a largest distance of 25 degrees (Fig. 3D). For all 29 penetrations, we presented the stimuli for which the RF fell on the center of the figure or on the ground, in 21 penetrations we also placed the edge in the RF, and in 22 penetrations we presented the “hole” stimulus without direct RF stimulation (Fig. 2E).

We first examined the overall activity by pooling activity across trials, cortical depths per penetration and then across all penetrations (Fig. 3A). To determine the significance of differences in activity between stimuli, we treated the average activities across trials and channels of a penetration as independent samples. The edge elicited a stronger response than the background in the late, sustained time-window (80–300 ms) (N = 21 penetrations, paired t-test for all penetrations for which we included the edge stimulus, t(20) = 13.7, p < 0.001), but not during the early window (N = 21 penetrations, t(20) = 2.01, p = 0.058).(Fig. 3B). Similarly, the figure stimulus elicited more activity than the background during the late response phase (Fig. 3B, right) but there was no difference in activity during the early response phase (0–80 ms) (Fig. 3B, left) (N = 29 penetrations, paired t-tests for all penetrations, early window, t(28) = 1.5 p = 0.144; late window, t(28) = 5.55, p < 0.001). We next determined how well neurons at individual MUA recording sites discriminated between the different stimuli at the single trial level by computing d’-values. During the late, sustained response phase, the average d’ for discriminating between edge and background was 0.79 (Fig. 3E) and it was 0.31 for the discrimination between the figure center and background (Fig. 3E). Note that the orientation of the gratings was balanced across the trials with a figure or ground in the RF so that the modulation of the responses is due to the figure-ground organization and does not reflect the orientation preference of the recorded neurons.

An unexpected effect was illustrated for the example penetration in Fig. 2E, where the “hole” condition, in which nothing appeared in the neurons’ RF, nevertheless elicited a delayed response. Comparable delayed responses driven from outside the classical receptive field have previously been observed in area V1 of monkeys^32^. We also investigated this effect across the population of penetrations and indeed observed a significant delayed response (N = 22 penetrations, paired t-test for all penetrations including the hole stimulus, t(21) = 4.5, p < 0.001; green in Fig. 3A) with a timing that was comparable to that of the figure-ground modulation, i.e. the difference in activity elicited by figure and ground (purple in Fig. 3A). Like the figure-ground modulation, the response to the hole stimulus represents a contextual influence on V1 activity from outside the neurons’ RF, because we ensured that the grating of the “hole” stimulus did not directly activate the RF (Fig. 3D). It may be related to the perception of a figure at the location of the hole, but we did not include the hole stimulus in our behavioral experiments so that this inference remains speculative.

### Analysis of latencies

We used a curve-fitting method to measure the latency of the visual response and the figure-ground modulation (described in Methods) (Fig. 3C; 20 penetrations with the relevant stimulus conditions). The average latency of the visual response was 52 ms, which was significantly earlier than the extra activity elicited by the edge with an average latency of 70 ms (p < 0.01; bootstrapping test, see Methods) and the figure-ground modulation with a latency of 72 ms (p < 0.01). There was no significant difference between the timing of the extra activity elicited by the edge or the center of the figure (p > 0.05).

### Activity of single units in V1 during texture segregation

We confirmed the MUA findings with an analysis of the activity of 85 single units, isolated with a spike-sorting method. For 59 of these cells, we also measured the activity elicited by the edge and the grey hole.

**Figure 4:**
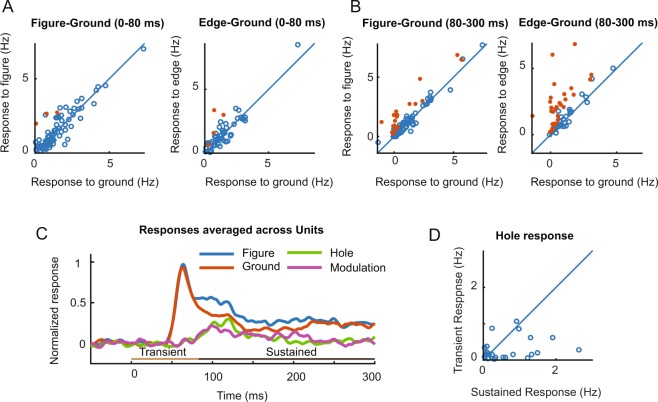
Figure ground modulation of single V1 neurons. (**A**) Comparison of firing rates of single units in V1 during the early time-window (0–80 ms) elicited by the figure center and background (left, N = 85), and elicited by the figure edge and background (right, N = 59). Red dots illustrate neurons for which the response differed significantly (p < 0.05). (**B**) Same as A, but during the later time-window (80–300 ms). (**C**) Average V1 response of single units (N = 59). Before averaging, the activity of individual neurons was normalized by first subtracting the baseline activity and then dividing by the peak response in the ground condition. The purple trace illustrates the time course of figure-ground modulation, which is the difference between the activity elicited by the figure and the background (i.e. blue minus red curve). The green trace shows the average response to the hole condition. The horizontal lines above the x-axis illustrate the early (light brown, 0–80 ms) and late time-windows (dark brown, 80–300 ms) used in the analysis. (**D**) Single unit response upon presentation of ground and hole stimuli during the transient (y-axis) and the sustained phase (x-axis) (N = 59).

Just as was the case for the MUA, the initial single unit transient responses (time-window 0–80 ms after stimulus onset) were similar irrespective of whether the figure or ground fell in the RF (Fig. 4A) (N = 85, paired t-test for all single units, t(84) = 1.2, p = 0.25), but we did observe that the edge elicited a slightly stronger response than the background (N = 59, paired t-test for all single units for which the edge stimulus had been presented, t(58) = 2.1, p = 0.04). As expected, V1 activity reflected figure-ground organization during the later, sustained response phase (80–300 ms, Fig. 4B). Now, the edge evoked more activity than the background (N = 59, paired t-test, t(58) = 5.6, p < 0.001), and the figure also evoked more activity than the background (N = 85, paired t-test for all single units, t(84) = 5.0, p < 0.001).

We presented the hole stimulus to control the placement of the figure in the receptive field of each cell recorded through the laminar probe. If the grating that surrounded the hole would fall in the receptive field, it should increase the neurons’ firing rate during the early response window. However, we did not observe such an early increase in the firing rate of the single unit population (N = 59, Fig. 4C,D). However, the hole stimulus did elicit a delayed response of the single units, with a strength and time course that was remarkably similar to the difference in activity elicited by the figure and ground (Fig. 4C). This delayed activation was significant for 25 of 59 single units (t-test, p < 0.05) (Fig. 4D). The response to the hole was also significant at the population level (N = 59, t(58) = 3.9, p < 0.001, Fig. 4D).

### The laminar profile of figure-ground modulation

**Figure 5:**
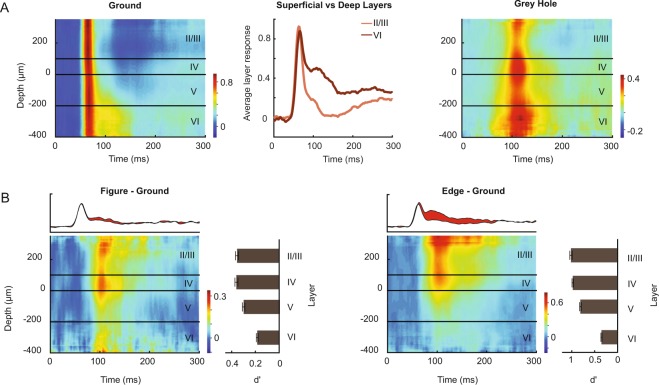
Laminar profile of figure ground modulation of V1 MUA. **(A**) Laminar profile of the MUA response elicited by a homogeneous grating (left) and by the stimulus with the grey hole (right). X-axis, time; y-axis, laminar depth. The middle panel compares the response evoked by the ground in the superficial and the deep layers. It can be seen that the activity is more sustained in the deep layers. (**B**) Laminar profile of the figure-ground modulation, i.e. the difference in activity evoked by the figure and the background (left) and the edge and the background (right). The average activity across the layers is shown in the panel above and the figure-ground modulation in red. Right panels, the average d′ values during the sustained response phase (80–300 ms) in the different layers.

The advantage of MUA over single unit activity is that we could measure reliable responses at most of the recording sites, which allowed us to examine the laminar profile of the V1 activity. We first examined the average laminar profile of visually driven activity by assessing the MUA response after aligning the depths of all penetrations according to their CSD profile elicited by the ground stimulus (see Methods) (Fig. 5A, left) and by then averaging the activity per cortical depth level across the penetrations. The transient response was nearly simultaneous across the layers and it can be seen that sustained firing rate was strongest in the deep layers, especially in layer 6, whereas the response in the superficial layers was more transient and suppressed thereafter (Fig. 5A, middle). We next examined the laminar profile of the figure-ground modulation, i.e. the difference between the response evoked by the figure and background (Fig. 5B, left panel) using a repeated measures ANOVA across 29 penetrations, with factor layer (L2/3, L4, L5 and L6) during the sustained response phase. There was a main effect of layer (F(1.36, 38.1) = 6.4, p = 0.009). Post-hoc tests revealed that the figure-ground modulation in layer 5 was weaker than that in layer 2/3 (p = 0.017) and layer 4 (p = 0.025). The difference in activity elicited by the edge and the background (Fig. 5B, right panel) followed the same pattern (F(1.76, 35.3) = 27.3, p < 0.001). Post-hoc tests revealed that the edge-ground modulation in layer 2/3 and layer 4 was stronger than that in layers 5 and 6 (all ps < 0.001), while the modulation in layer 6 was weaker than that in layer 5 (p < 0.01).

We also examined the laminar profile of the delayed responses elicited by an annulus stimulus (“grey-hole” Figs 2E and 5A, right). This activity occurred in all layers with a timing that resembled the timing of the figure-ground modulation. To examine possible differences between layers, we performed a repeated measures ANOVA. There was a main effect of layer (F(1, 21) = 8.7, p < 0.01) with weaker activity in layer 2/3 than in the other layers (all ps < 0.05). This laminar profile was different from that observed for the figure-ground modulation, which was relatively strong in layers 2/3 (Fig. 5B).

### The current-source density profile across the layers

**Figure 6:**
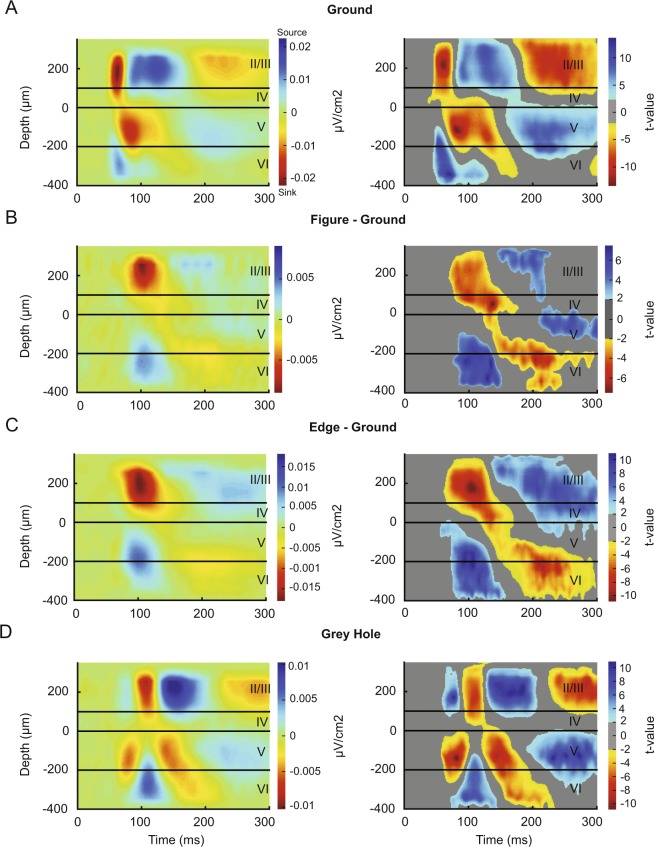
Analysis of the CSD. (**A**) left, CSD profile evoked by the background stimulus. Sinks are shown in warm colors and sources in cool colors, the values are in μV/cm2. The boundary between layer 4 and 5 was determined as the lower border of the early sink with a clear reversal visible at the depth marked as 0. Right, we determined the significance of the sinks and sources using a method based on clustering t-tests (see Methods). Significant (p < 0.05) sinks are shown in warm colors and significant sources in cool colors, the color indicates t-value. All sinks and sources that are visible in the left panel turned out to be significant. (**B**) Difference in the CSD profile evoked by the figure and the background. Note that the figure is associated with an extra sink in layer 2/3 that coincides with the increase in spiking activity. (**C**) The difference in the CSD between the edge and the background. The laminar profile is similar to that in panel B, but the edge elicits a slightly stronger sink in layer 2/3. (**D**) Laminar CSD profile evoked by the grating stimulus with a grey hole at the RF location. Note the initial sink in layer 5, which is followed by a CSD profile that resembles the one in panel B.

We calculated the CSD profile because current sinks in the CSD reveal where the current flows into neurons and hence the layers of putative excitatory synaptic inputs. To determine the visually driven synaptic inputs, we examined CSD evoked by the onset of the background grating in the neurons’ receptive field (Fig. 6A, left). To assess the significance of the sinks and sources, we used a method based on clustering t-tests (Fig. 6A, right) (Self *et al*. 2013). The visually driven response caused a strong and early sink in L2/3 and L4, which was followed by another sink in L5 (Fig. 6A). We next examined the difference between CSDs elicited by the figure- and ground stimuli to examine the location and time of putative synaptic inputs that are responsible for the extra spiking activity elicited by a figure (Fig. 5B). The figure caused an extra sink in L2/3 starting around 70 ms after the stimulus onset, which was accompanied by a source in layer 5. The timing of this sink coincided with the effect of figure-ground organization on the spiking activity. The CSD difference between edge and ground was almost identical to the difference between figure center and ground (Fig. 6C).

The CSD response elicited by the stimulus with a grey hole over the RF was different (Fig. 6D). It started with a sink in layer 5, where spiking activity also was relatively strong (Fig. 5A), suggesting that layer 5 may receive input from the surrounding cortical regions in V1 and/or higher areas representing the grating. Interestingly, this initial sink was followed by a sink in layer 2/3 that then moved down towards layer 5, resembling the difference between CSDs evoked by the figure and the background (Fig. 6B). The pronounced difference in the CSD between the background grating (Fig. 6A), which elicited the typical feedforward response from the LGN, and the hole stimulus (Fig. 6D), which did not, confirms that the hole was well centered on the RF (Fig. 3D) so that the grating did not infringe into the RF.

## Discussion

To our knowledge, the present results are the first that indicate that mice are able to detect and localize a figure defined by a difference in orientation from its background. Their ability to segregate a figure from the background was fairly robust to substantial variations in the size, position and grating phase, suggesting that it was not based on low-level perceptual strategies, like detecting luminance or an edge at a particular screen location. The most difficult phase in the training process of the mice was the transfer from the luminance defined figures to purely orientation defined figures. Accordingly, we had to adapt the training protocol to successfully train most of the mice to detect the orientation-defined figures. The ability to segregate figures from the background is important for aspects of natural mouse behavior, such as hunting for insects^22^.

Our results extend a recent study demonstrating that mice can detect the orientation of a grating if it is defined by variations in texture^33^ by showing that they can also localize objects defined by an orientation difference. They also complement studies in rats, which are able to recognize objects even if they have undergone transformations such as rotations, size changes or caused by different lighting conditions^16^. A previous study demonstrated that rats can also segregate and recognize orientation defined figures on a background with the orthogonal orientation^17^. Just as the mice of the present study, the rats also had initial difficulties to generalize from luminance defined shapes to orientation defined figure-ground stimuli. The finding that mice perceive orientation-defined figure-ground displays is encouraging, because it implies that this species can be studied to gain insight into the neuronal mechanisms underlying this fundamental aspect of visual perception.

We also measured activity in area V1 of awake mice when they saw orientation-defined figure-ground stimuli. When the background of the stimulus fell in the receptive field, it elicited a strong transient response that quickly decayed in the superficial layers but that was more sustained in the deep layers of cortex (Fig. 5A). A previous study demonstrated that such a quick decay of the visual response is particularly pronounced in awake mice and weaker when the animals are anesthetized^34^. When a figure fell in the neurons’ RF, it elicited more activity than the background. This figure-ground modulation was absent from the initial transient response elicited when the grating appears in the receptive field, but occurred during a later phase of the response. This finding is in accordance with previous results in awake monkeys^2,3,12^ and anesthetized mice^20^. The figure-ground stimuli were designed such that the stimulus in the neurons’ RF was the same, irrespective of whether it was part of the figure or background. Hence, the figure-ground modulation is an influence from beyond the classical RF, and the delay presumably reflects the polysynaptic routes associated with top-down effects from higher visual cortical areas and/or horizontal connections within V1^35^. In the present study, we presented relatively large figures with a size of 50°, which was approximately twice the size of the MUA RFs. These figures were more than 10 times larger than the figures used in previous studies in monkeys but we note that the V1 RFs in mice are also more than 10 times larger than those in monkeys, in accordance with their lower visual acuity^36,37^. It is remarkable that the neuronal mechanisms underlying figure-ground perception are so similar in these two species, given the large differences in acuity and also in the organization of the visual pathways. This similarity supports the view that figure-ground perception is a basic visual function, which has been preserved during the evolution of these species.

It is of interest that the grey hole stimulus, for which the grating was entirely outside the classical RF also drove many V1 neurons, but with a delay that was similar to the delay of the figure-ground modulation. The hole stimulus elicited a strong sink in layer 5, which suggest that synaptic inputs into this layer are responsible for the spiking activity. In contrast, the typical feedforward response, which is driven directly by the LGN, invariably started with a strong sink in layer 4. To our knowledge, this is the first characterization of the visual response elicited by such a hole stimulus in mice, without direct stimulation of the classical RF in V1. We suggest that the mice may have perceived the stimulus as a grey figure on a grating background, but did not test the perception of this stimulus in our behavioral experiments. Similar hole responses have been observed in monkeys^32^ and also with fMRI in humans where these contextual signals are informative about the stimulus that is presented outside the neurons’ receptive field^38,39^. Although the similarity of V1 activity evoked by figure-ground stimuli in mice and monkeys is remarkable, there are also a few differences. The first is the laminar profile. In the monkey, the enhanced activity elicited by a figure is most pronounced in the superficial and deep layers and it is weaker, but not absent, in layer 4^12^. A previous study demonstrated that figure-ground modulation in the anesthetized mouse is strongest in the superficial layers and in layer 4, but it was not yet known if this difference was caused by the anesthesia, as deep anesthesia abolishes figure-ground modulation in the monkey^40^. Here we found a laminar profile of figure-ground modulation in the awake mouse that is similar to that in the anesthetized mouse, with strongest modulation in layers 2/3 and 4 only weak modulation in layers 5 and 6. The laminar CSD patterns provide insight into the excitatory synaptic input responsible for the increase in spiking activity at the figure location. The figure gave rise to an extra current sink in the superficial layers that coincided with the increase in spiking activity, suggesting that extra synaptic drive in these layers is responsible for figure-ground modulation. In the monkey, however, the increase in spiking activity coincides with two sinks^12^. One sink occurs in the superficial layers and is similar to the sink in mice, but in the monkey a second sink occurs in layer 5, which is absent in the mouse. It is conceivable that this difference is caused by a difference in cytoarchitecture between these species. Layer 4 of mouse V1 is much thinner than layer 4 in the monkey (100 um compared to 500 um) and it is less distinguishable from the supragranular layers, because there are many direct connections from the LGN to the supragranular layers^41^. Furthermore, the laminar pattern of feedback connections to mouse V1^42^ may also be more diffuse than that in the monkey^43^. However, a species difference is not the only possible explanation. Another possibility is that the monkeys of the aforementioned studies carried out a task in which they had to make an eye movement to the figure, whereas during the recordings of the present study, the mice passively looked at the screen. We can therefore not exclude the possibility that figure-ground modulation might also occur in the deep layers of mouse V1 if they carry out a task in which they have to actively process the stimulus. Instead, we used freely moving mice to study their perception and we used a passive viewing task for our neurophysiological recordings. It would be very useful if future studies could develop paradigms for figure-ground perception in head-fixed mice so that researchers can study the neurophysiological signatures of figure-ground perception during active perception.

A second difference between studies carried out in mice and monkeys is in the response elicited by the edge between figure and background. In the monkey, the V1 response to a figure-ground stimulus is characterized by three successive phases^3,10,12^. The first phase is the visually driven response with a latency of ~40 ms. During the second phase with a latency of ~60 ms V1 activity starts to increase at the edges between figure and background, and during the third phase with a latency of ~90 ms figure-ground modulation also occurs at the center of the figure. In the mouse, we observed only two phases, because the latency of figure-ground modulation at the edge (70 ms) was almost identical to that at the center of the figure (72 ms) although the activity elicited by the edges was stronger, on average, than that elicited by the center of the figure. We note, however, that a genuine species difference is, once more, not the only possible explanation. The figure-ground displays used in the previous monkey studies were composed of many line elements with a width less than 0.1°, which is too small to be resolved by the mouse visual system. In the present study, we had to use grating stimuli instead, and to our knowledge it is not yet known whether the latency of the figure-ground modulation at the edge and in the center of a figure would differ in V1 of monkeys looking at these stimuli.

We conclude that mice perceive orientation-defined figures and that V1 neurons in the mouse enhance their activity if their receptive field overlaps with the figure. As a result, regions that belong to the figure are labeled with enhanced V1 activity, while activity in the ground regions is suppressed. An exciting next step would be to record while mice perform a task that requires figure-ground perception. This would require a figure-ground perception task in head-fixed mice so that the visual stimulus can be controlled more precisely. We expect that future studies can now take advantage of the many new methods that exist in mice to study the feedforward, lateral and feedback interactions between and within cortical areas^44,45^, the interactions between the cortex and subcortical structures and the role of interneurons^46^, to better understand the mechanisms underlying figure-ground segregation and, more generally, the processes that support image understanding in the vertebrate brain.

## Electronic supplementary material

Supplementary Information

## Data Availability

The datasets generated and analyzed for the current study are available upon request.
