# Supplementary material for: Figure-ground perception in the awake mouse and neuronal activity elicited by figure-ground stimuli in primary visual cortex
Source: Sci Rep. 2018 Dec 12;8:17800. doi: 10.1038/s41598-018-36087-8 (PMC6290763; doi:10.1038/s41598-018-36087-8)
Supplement: Supplementary file 1 — Supplementary Information [file 41598_2018_36087_MOESM1_ESM.pdf]

Figure-ground perception in the awake mouse and neuronal activity elicited by figure-ground stimuli in primary visual cortex – SUPPLEMENTARY INFORMATION

Ulf H. Schnabel[1,+,] , Christophe Bossens[2,+], Jeannette A. M. Lorteije[3], Matthew W. Self[1], Hans Op de Beeck[2,#], Pieter R. Roelfsema[1,4,5,#,*]

[1]Department of Vision & Cognition, Netherlands Institute for Neuroscience, Meibergdreef 47, 1105 BA, Amsterdam, The Netherlands

[2]Laboratory of Biological Psychology, Brain & Cognition, KU Leuven, Leuven, Belgium

[3]Faculty of Science, Swammerdam Institute for Life Sciences, University of Amsterdam, Amsterdam, The Netherlands

[4]Psychiatry Department, Amsterdam University Medical Center, University of Amsterdam, Amsterdam, The Netherlands

[5]Department of Integrative Neurophysiology, Vrije Universiteit, Amsterdam, Amsterdam Neuroscience, The Netherlands

[+]Co-first Authors

[#]Co-last Authors

[*]Corresponding Authors

CORRESPONDENCE

P.R. Roelfsema, e-mail p.roelfsema@nin.knaw.nl, phone +31-20-5664587, fax +31-20-5666121

SUPPLEMENTARY INFORMATION – Learning progression

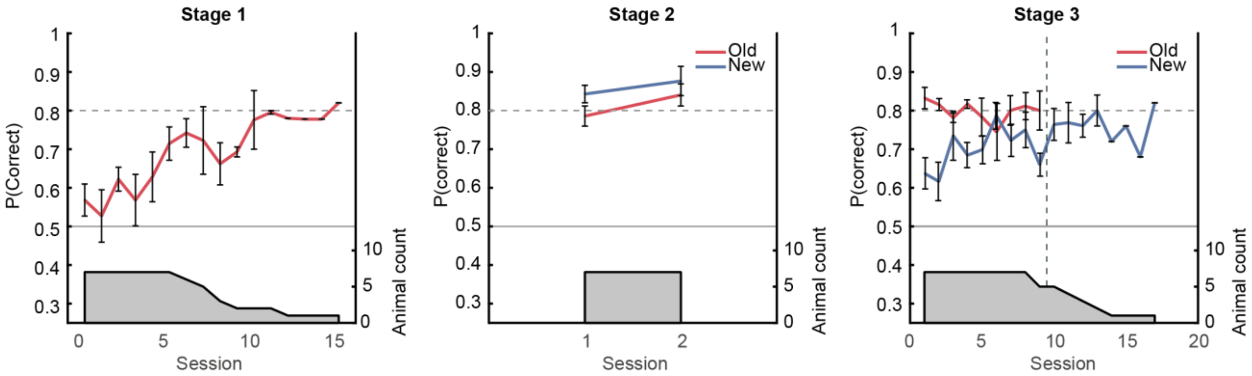

Figure S1. Progression of learning through the three stages. Learning curve across sessions with stage 1 (left), stage 2 (middle), and stage 3 (right) stimuli. During stage 2 and stage 3, performances are shown separately for old stimuli (i.e., all the stimuli that had been learned in the previous stage) and new stimuli. In the initial phase of stage 3, animals saw both old and new stimuli, but after 9 sessions we only presented new stimuli, as indicated by the vertical dotted line. The animals progressed to the next stage when they reached criterion performance. The lower panel illustrates the total number of animals in a particular stage (right y-axis).
